# DNA metabarcoding to estimate diet overlap between the introduced Joro spider (*Trichonephila clavata*) and three native orb-weaving spiders

**DOI:** 10.1371/journal.pone.0351929

**Published:** 2026-06-24

**Authors:** Erin E. Grabarczyk, Jason M. Schmidt

**Affiliations:** 1 Department of Biology, Valdosta State University, Valdosta, Georgia, United States of America; 2 Department of Entomology, University of Georgia, Tifton, Georgia, United States of America; Charles University: Univerzita Karlova, CZECHIA

## Abstract

The introduction of novel generalist predators to new ecosystems can dramatically alter species interactions and established food webs. Invasive predators may contribute to pest control services; however, a net loss of biodiversity can occur if invasives displace natives through intraguild predation or resource competition. Joro spiders (*Trichonephila clavata*) are an introduced, orb-weaving spider that show rapid range expansion in the United States. Trophic patterns of orb-weaving spiders are largely unknown, and as such, the impact of Joro spiders on established food webs is unclear. We explored patterns of diet composition and prey overlap between Joro spiders and three co-occurring, native orb-weaving species with molecular gut content analysis. In addition, we asked whether the composition of focal native spider diets differed at sites co-inhabited by Joro spiders. We collected female spiders from 52 sites within the Joro spider’s introduced range and analyzed gut content via DNA metabarcoding and high-throughput Illumina sequencing. Despite overlap in many prey taxa consumed, overall diet composition was dissimilar between Joro and native spiders. Joro spider diets were distinct, with at least 26 unique prey taxa not detected in native spider diets. Moreover, native spider diets were similar regardless of whether Joro spiders were present at collection sites. Thus, our initial analysis suggests that while Joro spider diets do overlap with native spiders, their use of many unique food resources does not suggest strong competition. Additional research into web placement and spatial overlap as mechanisms underlying Joro spider invasion success should help clarify the potential for exclusion of native predator populations.

## Introduction

Invasive generalist predators change the trophic structure of established communities, altering species interactions [1,2]. While invasive predators may contribute to pest control services, a net loss of biodiversity occurs if they displace native species through intraguild predation or competition over food resources [1,3,4]. As a result, prey resource competition may shrink diet breadth or shift diet composition of native predators [5]. Following an invasion, identifying change to food web structure is critical but can be challenging to carry out in real time [6]. Molecular analysis with DNA metabarcoding allows for identification of trophic niche overlap in diverse taxa including birds [e.g., 7,8], mammals [e.g., 9–14], fish [e.g., 15], and arthropods [e.g., 16,17]. Estimating trophic interactions with molecular analyses is advantageous, as this approach generates a relatively quick snapshot of consumer diet composition and diversity through the construction of food webs [18–21].

Orb-weaving spiders are common generalist predators that play fundamental roles in a variety of terrestrial ecosystems [22,23]. Globally, many orb-web species have been introduced outside their native range [24–26]. Unlike highly mobile generalist predators, orb-weaving spiders sit and wait for prey to be trapped on webs. Due to the nature of prey capture, resource competition between species differs from other, more mobile generalist predators. Consequently, newly introduced spiders tend to compete with natives or other established introduced species over web placement [25,27–29] or negatively impact native populations through intraguild predation [27,30,31]. Despite many examples of spider invasions, to date, limited evidence supports competitive exclusion due solely to trophic overlap between invasive and native orb-weaving spiders [28]. However, as novel species introductions increase, we should seek to understand the patterns of trophic overlap as well as the ecological mechanisms that contribute to invasion success.

The Joro spider (*Trichonephila clavata*) (Koch 1878) was first confirmed in the southeastern United States during 2014 [32], and modeling suggests the potential for significant range expansion [33–35]. In their native and introduced range, Joro spiders thrive in forests as well as urban areas [36,37], possibly due to physiological adaptations to high-stress environments [38,39]. Joro spiders exhibit extreme sexual size dimorphism, with females measuring more than three times the size of males in their introduced range [40]. In autumn, female Joro spiders spin massive, golden webs to capture prey. Field observation of web foraging behavior and initial molecular gut content analyses indicate that a large proportion of Joro spider diets consist of small flies [40,41]. Females also consume Hemiptera, Hymenoptera, and Coleoptera [40–42]. However, whether the diet of Joro spiders overlaps with native orb-weaving spiders within their introduced range is unclear.

The phenology of several native orb-weaving spiders coincides with that of the introduced Joro spider. For example, *Neoscona crucifera* (Lucas, 1893) and *Araneus marmoreus* Clerck, 1757 are nocturnal orb-weaving spiders that are slightly smaller than Joro spiders [43,44]. *Neoscona crucifera* spin a new web every night in a variety of habitats including both forest and suburban areas [43,45], plastically adjusting the size of their web depending on perceived prey availability [45]. *Araneus marmoreus* build webs in bushes, grasses, or the lower limbs of trees [43,46]. Smaller, forest-dwelling, orb-weaving spiders include two diurnal species, *Gasteracantha cancriformis* (Linnaeus, 1758) and *Micrathena mitrata* (Henz, 1850) as well as the nocturnal *Verrucosa arenata* (Walckenaer, 1841) [43,44]. *Gasteracantha cancriformis* build webs in a variety of habitats including forest, agriculture, and suburban areas [43,47]. Thus, several species of native orb-weaving spiders may overlap with the Joro spider within their introduced range.

Since their introduction to the United States, many researchers have suggested Joro spiders may limit native orb-weaving spider populations due to trophic niche overlap [34,44,48]. Although complete diet overlap is unlikely due to many differences in species’ life histories, currently, we lack basic information, such as diet composition of native orb-weaving spiders, in areas where non-native Joro spiders are now found. Therefore, to advance our understanding of Joro spider biology within their introduced range, we characterized their diets with molecular gut content analysis. In addition, we collected native orb-weaving species that occupy the same microhabitats as Joro spiders. Our goal was to take the first, initial steps to understand patterns of diet overlap between Joro spiders and native orb-weaving species. To achieve this, we collected female spiders from their webs and used molecular gut content analysis to reveal dietary trends in arthropod prey taxa. Specifically, we tested 1) species-specific diet metrics including richness (niche breadth) and composition, 2) diet overlap between the Joro spiders and native orb-weaving spiders, and 3) whether the diet of native spiders differs in areas co-inhabited by Joro spiders and in areas where Joro spiders were not recorded. We predicted that based on similar foraging modes (i.e., orb-weaving species), the diets of focal species would show considerable overlap. However, because of species-specific traits (e.g., color, size, behavior), each species likely consumes unique prey taxa not detected in the diets of other focal spiders.

## Methods

### Sample collection and spider identification

During October 2, 2021 – October 24, 2021, we collected 356 female orb-weaving spiders from 52 sites in Georgia and South Carolina, United States (S1 Fig in S1 File). We selected 49 public access parks to hunt for spiders based on iNaturalist reports of Joro spiders in the region as well as parks just outside the confirmed Joro spider range. In addition, we sampled three private residences where the presence of Joro spiders had been confirmed. Permission was granted by homeowners to collect spiders from private property. No permits were required for field collection. All sites were separated by at least 4 km. At each site, we traversed edge habitat for approximately 30 minutes, searching for and collecting both female Joro spiders as well as native orb-weaving spiders that occurred in the same microhabitats. We collected orb-weaving spider species based on [44] as species that may compete with Joro spiders for prey items, that actively built webs in very close proximity to Joro spiders or in microhabitats where we would expect to find Joro spiders, and were mature adults during the time of collection. We focused on female spiders, as body condition and foraging studies suggest sexual dimorphism in feeding ecology, where females spend more effort feeding to generate energy for reproduction [e.g., 49]. And this pattern appears to be consistent in Joro spider feeding ecology as well [40]. Further, while males are relatively easy to find within Joro spider webs [40], we found zero males in or around the webs of native spiders. Captured females were placed in 20 mL sterile vials and submerged in 100% chilled ethanol. Specimens were kept on ice until transfer to a −20°C freezer for storage. In the laboratory, JMS identified native female spiders to species under a microscope according to [50].

We collected 213 female Joro spiders from 33 out of the 52 sites sampled and collected native orb-weaving spiders from 41 out of the 52 sites. For native spiders, we collected a total of 47 *Gasteracantha cancriformis*, 41 *Neoscona crucifera,* 26 *Araneus marmoreus*, 17 *Verrucosa arenata,* eight *Neoscona spp.,* and four *Micrathena mitrata*. We restricted our analysis of diet to native spider species with minimally 20 samples to provide the best replication possible given low numbers of native spiders collected (i.e., *A. marmoreus, G. cancriformis*, and *N. crucifera*). Moreover, we focus on native spiders that are likely meaningful competitors based on diel pattern, body size, and phenology [44] and that occupied the same microhabitats as Joro spiders.

### Metabarcoding and sample processing

We followed best practices to limit external contamination as well as contamination between samples during processing. First, to reduce the amount of focal predator DNA in our extractions, we dissected the intestinal tracts of all female spiders [51]. Dissected guts were saved in 100% ethanol at −20°C until DNA extractions. We homogenized spider gut samples then extracted DNA with the DNeasy Blood & Tissue Kit (Qiagen) according to the manufacturer’s instructions. Extractions were stored at −20°C until PCR preparation. Our negative controls (n = 4) included blank extractions of molecular grade water. For DNA metabarcoding, we followed a two-step PCR approach [40], and amplified samples via PCR with TruSeq TelperionF-LaureR spider exclusion primers, which targets an insert within the Folmer region of the *COI* gene [52]. The TelperionF-LaureR primers were designed, and previously optimized, to reduce spider amplification and favor insect DNA templates within samples providing a ~ 300 bp amplicon [44]. Next, we labeled each individual spider PCR product with Illumina adapters and dual-indexed barcodes, which included unique 10 bp index sequences for i5 and i7 (xGEN UDI Primer Pairs, Integrated DNA Technologies, Inc.). After both rounds of PCR, we cleaned products with magnetic beads (MagBio HighPrep^TM^ PCR System) and screened samples on a QIAxel Advanced system (Qiagen). Samples were pooled based on their concentrations, combined with 30% PhiX added to the final pool, and sequenced on an Illumina NextSeq 2000 (Illumina, San Diego, CA, USA) with NextSeq 1000/2000 P2 reagents (600 cycles) by the Georgia Genomics and Bioinformatics Core laboratory (GGBC-UGA).

### Read processing and taxonomic assignment

For bioinformatics, we followed [40], with current standard practices and software. Briefly, raw sequence data was first demultiplexed with pheniqs v2.1.0 [53], enforcing a strict match of sample barcode indices. To separate raw reads into per-sample FASTQ files, we ran a second round of demultiplexing with pheniqs using MIDs located at the beginning of the paired insert reads. Inline MIDs were trimmed during demultiplexing, which left the target amplicon. Cutadapt v3.4 [54] was used to remove gene primers from the forward and reverse reads and discarded read pairs where one or both primers were not found at the expected location (5’) with an error rate less than 0.15. We merged read pairs with *VSEARCH* v2.15.2 [55] and discarded resulting sequences less than 150 bp and greater than 400 bp in length, or with a maximum expected error rate greater than 0.5 bp [56]. Next, we clustered reads for each sample using the unoise3 denoising algorithm [57] as implemented in *VSEARCH*, setting an alpha value of five and discarding unique raw sequences observed less than eight times. In *VSEARCH,* we compiled counts of the resulting Exact Sequence Variants (ESVs) and removed chimeras with the uchime3 algorithm. For all final ESVs, we assigned a consensus taxonomy according to a best-hit algorithm and the GenBank publicly available sequence reference database. For reference database searching, we used an exhaustive semi-global pairwise alignment with *VSEARCH*. We quantified match with a custom, query-centric approach, where the percent match ignores terminal gaps in the target sequence, but not the query sequence. We created a consensus taxonomy with either all 100% matching reference sequences or all reference sequences within 1% of the top match, accepting the reference taxonomy for any taxonomic level with greater than 90% agreement across the top hits. Finally, we followed a stringent multi-method process to remove overall low-frequency reads, low frequency taxa, and contamination in negative controls [40,42]. For our negative controls, we processed a blank DNA extraction, with no animal tissue added to the reagents, then included this extraction as a sample during PCR all the way through pooling of libraries and sequencing [58,59].

### Statistical analysis

We ran a series of models with R program software version 4.5.2 (R Core Team 2025) to evaluate diet metrics and overlap between female Joro and native orb-weaving spiders. To analyze prey diversity and richness, we fit rarefaction curves to estimate prey diversity in relation to the number of spider gut samples collected and to compare between spider species (package: *iNext*) [60,61]. For comparisons of diet composition, we reduced the taxa detected in spider guts to the common taxa to aid convergence of multivariate models and reduce bias of rare taxa likely detected at higher probabilities in species with more sampling effort [62]. We estimated differences in prey taxa detected to centroids with a permutation test with a sample-size bias adjustment (package: vegan, function: ‘betadespers’) and conducted a PERMANOVA to test for significant dissimilarity in prey taxa detected between spider species with strata set at collection site [63] (package: *vegan*, function: ‘adonis2’). Next, we refit both analyses to estimate differences in native species diets depending on whether Joro spiders were found at sites during sampling. For both dissimilarly analyses, we projected our results using PCoA to display adjusted median centroid dispersion in prey DNA detected in spider taxa as well as by NMDS.

To aid interpretation of dissimilarity and diet overlap results, we used an indicator species analysis to determine significant associations between commonly detected prey taxa by spider species, weighted across species based on sample size [64] (package: *indicspecies*, function: ‘multpatt’). To visualize diet overlap according to spider species, we generated a Venn diagram to show unique prey taxa as well as formed rank abundance displays to highlight change in rank abundance of the top 10 most common prey taxa detected by spider. Finally, to determine the amount of diet composition overlap among spider species, we calculated the Pianka index for pairwise correlations and the Levin index to estimate niche width [15] (package: *spaa*). We conducted all analyses at the level of family.

## Results

### Summary of sequencing read depth and sample processing

Arthropod prey consumed by spiders successfully amplified with the TelperionF-LaureR spider exclusion primers. Overall, we recovered a total of 94,699,073 high quality reads (>Q30, greater than 150 bp and less than 400 bp overlap, with greater than 90% taxa match at the family, genus, and species level). Due to a considerable proportion of the total taxa assignments detected at the family level (8,460 out of 10,735), all reads were combined at the family level. Although the TelperionF-LaureR primers are designed to limit amplification of spider DNA, a significant number of reads matched each of the focal spider species (Table 1). Therefore, we took a conservative approach and removed all Araneae reads from our samples for analysis and included the remaining reads as estimates of prey DNA detection (an indirect proxy for diet composition). Of our four negative controls, one control contained arthropod reads (total of 27 reads). Therefore, we used a positive threshold (Mean ± 1 SE) equivalent to 20.25 and did not consider taxa that contained less than or equal to 21 reads. We recovered 27,731,115 total reads for non-spider arthropod taxa, which resulted in an initial dataset representing 29% of the total sequence reads as insect prey DNA detected in spider gut samples.

**Table 1:** Summary of overall sample sequencing depth represented as number of total reads, mean Aranea (spider) reads, mean prey taxa recovered (±1 SE) as well as diet indices calculated based on DNA metabarcoding of spider gut contents. Estimates of prey DNA detection richness for each taxon were derived from rarefaction, and trophic niche width was estimated with Levin’s index.

Spider species	Total reads (1SE)	Araneae reads (1SE)	Prey taxa reads (1SE)	Observed prey richness (1SE)	Estimated prey detection richness Est. (LCL, UCL)	Niche width
*T. clavata*	161,756.3 (106,752.5)	59,981.9 (65,130.8)	101,774.4 (98,888.3)	156 (15.1)	206 (176, 235)	44.07
*A. marmoreus*	207,516.2 (122,104.6)	205,355.8 (122,190)	2,160.4 (4,507.9)	46 (15.5)	75 (46, 105)	34.93
*N. crucifera*	233,895.5 (306,091.1)	230,970.2 (307,695.2)	2,925.3 (17,297.3)	29 (12.5)	41 (29, 65)	23.31
*G. cancriformis*	356,559.1 (141,664.2)	356,311.7 (141,677.4)	247.4 (573.9)	22 (19.4)	38 (22, 76)	14.72

### Spider diet composition

**Fig 1:**
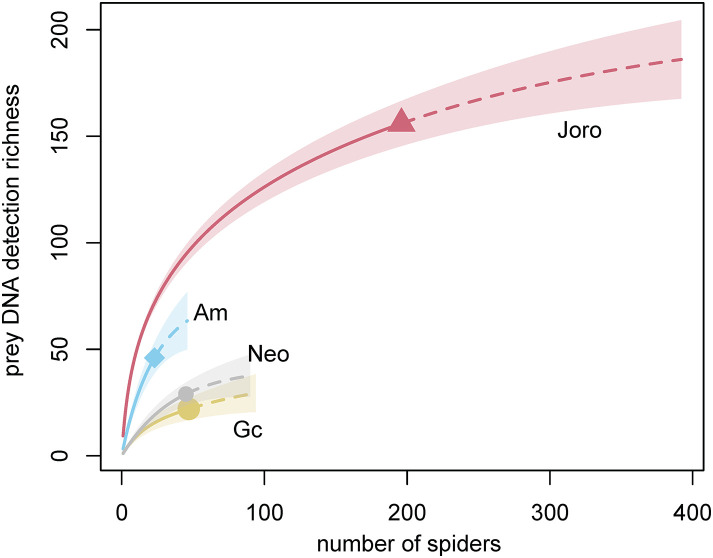
Rarefaction curve to estimate DNA detection richness of prey taxa in spider gut content samples for focal orb-weaving spiders. Estimated DNA prey detection richness for each curve was generated from hill number q = 0 (Table 1). Labels for spider taxa: Joro = *Trichonephilia clavata*, Am = *Araneus marmoreus*, Neo = *Neoscona crucifera*, Gc = *Gasteracantha cancriformis*.

Across all Joro spider and three native species samples, we detected 164 prey taxa, with most reads (i.e., relative abundance) classified as Diptera taxa (S1 Table). As expected, estimated prey richness was related to the total number of spider samples collected by species, and estimates from rarefaction were then used for richness estimates of prey detection (Fig 1, Table 1). According to rarefaction curves, sample coverage for Joro spider diet richness was high, with approximately 97% of taxa included in our prey richness estimates. For the native orb-weaving spiders, sample coverage was quite low, at 46, 22, and 29% (*A. marmoreus, N. crucifera,* and *G. cancriformis*, respectively). We collected the highest number of Joro spiders, and on average, using standardized estimates from rarefaction we predicted their diets to have the highest richness of detected prey, and the broadest niche width of the spiders collected. However, we show modest standardized estimates of prey dietary richness for the native orb-weaving spiders (Table 1), likely due to much smaller sample sizes.

### Spider diet dissimilarity and overlap

**Fig 2:**
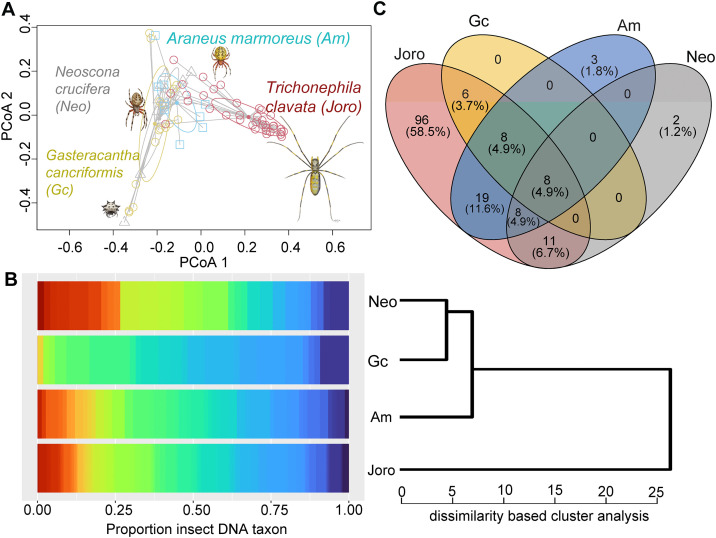
(A) PCoA of DNA prey detection by focal spiders to visualize diet composition dissimilarity with circles representing 95% CI around the centroid and center solid symbol represents the centroid for respective taxa by color. **(B)** Stacked bar combined with cluster analysis to show clustering of dissimilarity in diet detection between spiders. Colors represent a spectrum of prey DNA detected in spider gut samples. **(C)** Venn diagram displaying diet overlap of prey taxa by spiders with numbers (counts) within the area unique to each taxa and percentages of respective overlap. Labels for spider taxa: Joro = *Trichonephilia clavata*, Am = *Araneus marmoreus*, Neo = *Neoscona crucifera*, Gc = *Gasteracantha cancriformis*. Joro spider illustration by Noah Smith. Native spider illustrations by Steve Buchanan with permission from the American Arachnological Society.

One objective of our study was to identify aspects of dietary overlap between the introduced Joro spider and native orb-weaving spiders. To accomplish this and to adjust for low sample sizes, we reduced our dataset to common taxa detected in a minimum 5% of all samples, which resulted in 52 common prey taxa (S2 Table). Consistent with overall patterns of prey reads, Diptera represented 27 out of 52 common prey taxa (S3 Table). Between spider species, diet composition was dissimilar, based on a permutation test of sample size bias corrected distances of group centroids (F_3,75_ = 15.825, *P* = 0.001, n.perm = 999; Fig 2A, S2A Fig in S1 File). And the composition of native orb-weaving spider diets overlapped more so with one another than with Joro spiders (Fig 2B). Our combined analyses estimate common prey taxa detected in Joro spiders was 10–25% dissimilar, depending on analysis, to that of the three native orb-weaving spiders, which were at most 7% distinct (S2B Fig in S1 File).

### Indicator species analysis

**Fig 3:**
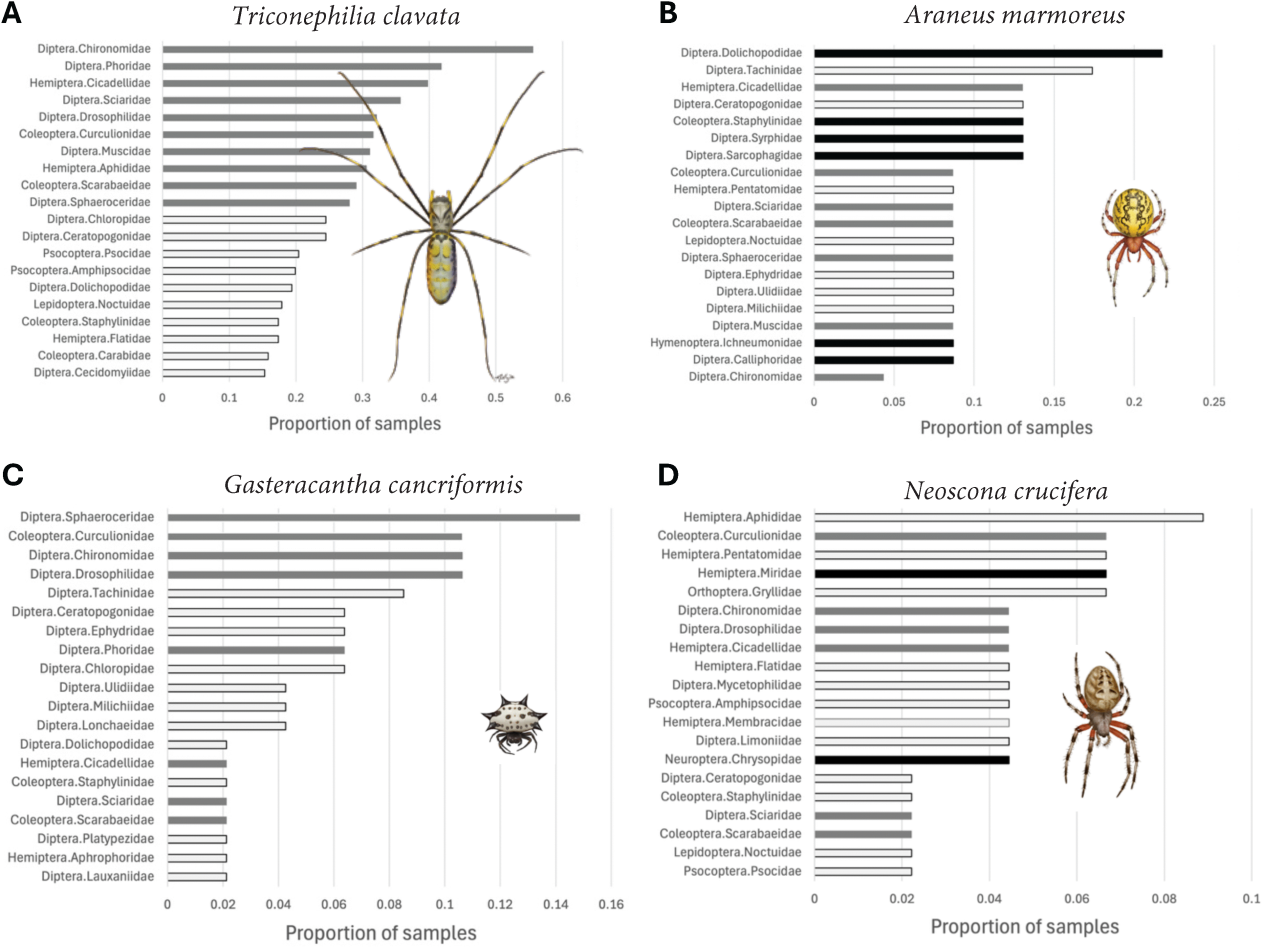
Prey rank abundance detected in orb-weaving spiders via DNA metabarcoding. Values represent the rank detection frequency of prey taxa in spider samples for each species. The gray bars indicate the top 10 most common prey in Joro spiders across panels to compare the ranks between spider taxa. White bars indicate either prey taxa outside the top 10 most common in Joro spiders or for other taxa for the spider in panel. Black bars illustrate significant indicator prey detection for native orb-weaving spiders. All rank abundance calculations are based on frequency of detection (FOO) of prey DNA by focal spider species. Joro spider illustration by Noah Smith. Native spider illustrations by Steve Buchanan with permission from the American Arachnological Society.

In addition to dissimilarity in the patterns of prey taxa detected between spider taxa, we found Joro spiders fed on many unique taxa (Fig 2C). We used an indicator species analysis to assess uniqueness in prey DNA detection, which suggests that Joro spiders ate 26 unique prey taxa at the level of family (11 Diptera, seven Hemiptera, three Coleoptera, two Pscoptera, one Lepidoptera, one Hymenoptera). *Araneus marmoreus* foraged on six unique prey taxa (four Diptera, one Hymenoptera, and one Coleoptera). *Neoscona crucifera* consumed one unique prey taxa (Neuroptera) and one additional taxon that was different between Joro spiders and *N. crucifera* (S1 Table). To further understand the differences between native and Joro spider diets, we formed rank abundance displays to highlight the change in rank abundance of common prey taxa detected by spider species (Fig 3). Thus, we compared the top 10 most common prey taxa as additional indicators, which illustrate differences in prey taxa consumed in each respective spider species (Fig 3). We calculated the Pianka Index (ranging from zero or no overlap to one or complete overlap) to evaluate pair-wise diet overlap between each spider species. We found a consistent diet overlap (approximately 0.6) between native orb-weaving spiders and the Joro spider, and the extent of dietary overlap depended on whether we estimated overlap with frequency of occurrence or read abundance (Table 2). Diet overlap estimates were higher when we calculated the Pianka index with frequency of occurrence estimates (i.e., presence or absence of prey taxa) compared to mean sequence reads by prey taxa. However, the overall patterns across the various diet analytical approaches suggested uniqueness in the prey DNA detected between Joro spiders and native orb-weaving spiders observed in this study.

**Table 2:** Diet overlap estimated with the Pianka index, calculated for each spider species according to presence or absence of prey taxa (top values) as well as mean reads by prey taxa (bottom values). Values range from zero or no overlap to one or complete overlap.

Spider species	*T. clavata*	*A. marmoreus*	*N. crucifera*	*G. cancriformis*
*T. clavata*	–	0.661	0.613	0.681
*A. marmoreus*	0.239	–	0.326	0.515
*N. crucifera*	0.323	0.048	–	0.301
*G. cancriformis*	0.404	0.096	0.273	–

### Effects of Joro spider presence on native spider diets

**Fig 4:**
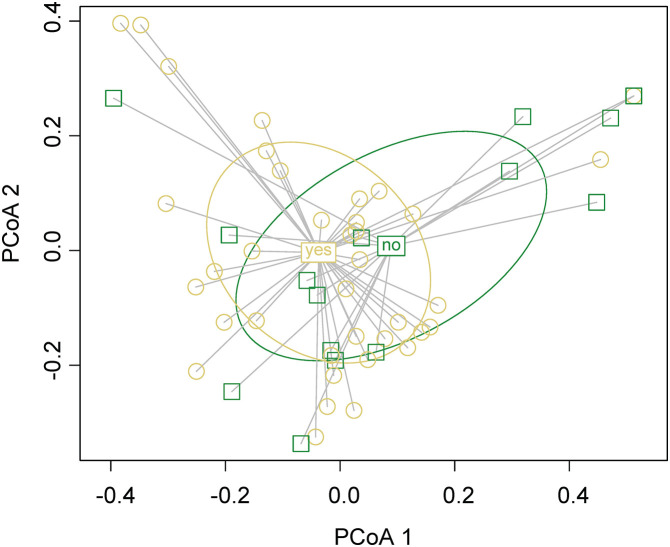
PCoA to visualize potential dissimilarity in prey DNA detection of native orb-weaving spiders combined at sites where Joro spiders were present (or yes, symbolized with yellow circles) or absent (or no, symbolized with green squares).

Finally, to determine whether the diets of native orb-weaving spiders shift or shrink when Joro spiders are present, we categorized each collection site by the presence or absence of Joro spiders at the time of sampling. Our initial results suggest the composition of prey taxa detected in native spiders does not significantly correlate with the presence of Joro spiders, based on a permutation test of sample size bias corrected distances of group centroids (F_1,52_ = 2.42, *P* = 0.14, n.perm = 999; Fig 4; S3 Fig in S1 File). That is, native spider diet composition was not distinguishably different when Joro spiders were present.

## Discussion

Through a molecular gut content analysis approach combined with a multi-faceted, multivariate dispersion analysis, we found introduced Joro spiders, *Trichonephilia clavata*, to have distinct diets compared to three co-occurring, native orb-weaving spiders. Importantly, diet composition of each spider species was unique, with some prey taxa detected only in respective species. Joro spiders in particular consumed at least 26 unique prey taxa that were not detected in native spider diets. In addition, our initial analysis of this system suggests that the composition of prey taxa detected in native spider diets was not altered by the presence of Joro spiders inhabiting the same location. However, given approximately 10–25% diet dissimilarity across multiple analyses, not unexpectedly, our results also suggest some overlap of prey taxa consumed among spider species. Thus, our initial analysis suggests that while we did find diet overlap between the Joro and native spiders, their diets were dissimilar, and at this time, Joro spiders are unlikely to outcompete the focal native spiders based on diet overlap alone. Generally, trophic niche partitioning in generalist spiders may be influenced by body size, hunting mode, and foraging stratum [65,66]. Other factors such as web architecture, body color, and behavior on the web require further attention in systems where orb-weaving species exhibit temporal and spatial overlap.

In our study, all spiders consumed small prey; some larger prey taxa were detected, which may be the result of rare prey types intercepting webs or prey preference [67]. In their native range, observation of female Joro spiders show that diets consist mostly of small prey (mean length = 1.95 mm), and much of their time foraging was spent on small prey [41]. Large prey taxa (>6 mm) consumption was rare [41]. Correspondingly, our data on DNA detection from Joro spiders in the United States show small Diptera, especially Chironomidae, as the most common prey taxa detected in both male and female Joro spiders [40] as well as the most common prey taxa detected from prey remains and fecal samples [42]. With the data presented here focusing on female gut content, we detected a high proportion of Diptera and estimated the total diet richness of over one hundred taxa at the level of family.

Overall, we anticipated the diet of our focal species to show some overlap, simply because all focal spiders capture prey on webs and occupy similar microhabitats. However, we also expected diet dissimilarity between orb-weaving species because of known differences in foraging strategies and behavior. For example, like other Nephilid spiders, brightly colored female Joro spiders sit at the center of large, golden webs [68] and freeze in response to disturbance [69]. The conspicuous behavior of sitting in a central web location and being colorful may act as a means to attract certain types of prey [70]. *Araneus marmoreus* is also a very strikingly colorful spider, however, this species retreats from disturbance [69]. Prey capture by *A. marmoreus* varies depending on web placement height [71] and web silk properties [72]. And while *Araneus marmoreus* may prefer larger prey, females opportunistically consume small prey caught in webs when recycling web material [73]. Thus, although Joro spiders and *A. marmoreus* are both colorful, may overlap in size and microhabitat web placement, their behaviors on the web likely led to the observed differences in diet composition. On the other hand, *Gasteracantha cancriformis*, the smallest of our focal species, also sits in the center of webs and is generally one color, yellow or white, with some patterning. Despite much smaller body sizes of *G. cancriformis* compared to the other three focal spiders in our study, this species was the only spider that did not consume unique prey taxa. Additional work should seek to quantify the amount of prey consumed by *G. cancriformis* compared to other spiders that occupy the same habitats to determine how vulnerable this species is to competitive exclusion. Thus, at first glance, we observed a substantial amount of diet overlap between spiders. However, graphical and indicator analysis of common prey taxa shows unique diet composition amongst our focal species, which may be due to small, nuanced differences in foraging tactics, behaviors, web properties and placement.

To the best of our knowledge, little information is available regarding the diet composition of the native orb-weaving species included in this study. Observation of *Gasteracantha cancriformis* foraging in citrus groves found this species consumes Diptera, Lepidoptera, and Coleoptera, and does not preferentially consume prey captured on webs [47]. Based on our molecular gut content analysis, the top prey consumed by *G. cancriformis* consisted mainly of Diptera followed by Coleoptera and Hemiptera. *Gasteracantha cancriformis* was the only focal spider in our study that did not consume unique prey taxa and had the smallest diet niche width (Fig 3, Table 1), which is in part due to the small number of *G. cancriformis* collected here. In France, observation of *A. marmoreus* webs suggests this species consumes large quantities of Diptera followed by Hymenoptera, Lepidoptera, Coleoptera, Hemiptera, and Orthoptera [71]. Here, *A. marmoreus* foraged on a variety of Diptera and Coleoptera taxa, several of which were indicator species and unique to this spider (Fig 3, S3 Table). In contrast to the other focal spiders, *Neoscona crucifera* ate a high proportion of Hemiptera, Coleoptera, and Orthoptera (Fig 3). *Neoscona crucifera* fed on Diptera but this was not the main prey taxa detected by gut content analysis. Although numerous prey taxa were recovered from native orb-weaving spider guts, our characterization of the total diet richness is conservative due to our small sample sizes [74]. This, however, was intentional, as we did not want to negatively influence native spider population sizes, particularly in areas co-inhabited by Joro spiders. We did not observe significant dissimilarity of common prey taxa detected across spiders in areas with and without Joro spiders, which suggests the presence of Joro spiders did not significantly impact diet composition (Fig 4). However, given low sample sizes for natives and considerable variability in prey taxa composition detected (Fig 4), additional gut content sampling of the focal native spiders may indeed uncover more unique prey taxa. Therefore, our dissimilarity estimates are conservative and focus on common taxa for which we have better sampling effort confidence [74]. For future study of the native spiders, collecting dietary samples with a non-lethal approach, such as fecal samples, may provide sufficient information without the need for destructive sampling [42,75].

Complete trophic niche overlap amongst generalist predators is uncommon [76]. And prey resource competition may not be the only mechanism that leads to biodiversity declines [3]. Displacement of native orb-weaving species may occur when an introduced species outcompetes others for web placement, aggressively takes over webs, or through intraguild predation. For example, the invasive European sheet-web spider, *Linyphia triangularis*, overtook webs of the native *Frontinella communis* [27]. However, successful takeovers often resulted from *F. communis* fleeing the web, and aggressive interactions were rare. Alternatively, *Pholcus phalangiodes* adjusted aggressive behavior when introduced to the webs of *Pholcus manueli* depending on the size of their competitor and body condition [29]. When artificially placed in close proximity, female Joro spiders attack and cannibalize other female Joro spiders [77]. However, such attacks in the wild are likely infrequent and whether Joro spiders aggressively take over heterospecific webs is unclear. With the current molecular approach, we did not attempt to differentiate between focal predator DNA with that of other spiders in the Araneae order. Despite using primers designed to exclude Araneae amplification [52], Araneae reads were present in all samples. Additional field observations are necessary to determine the frequency of web takeovers and predation events between Araneae spiders.

In general, spider foraging behaviors and dietary patterns are understudied, especially for species that occur outside of agroecosystems. Nevertheless, improved synthesis of spider traits and functions [78] show that, in general, web-building spider diets are more diverse compared to hunting spiders [79]. And spider webs may serve as an extended phenotype, as web-building spiders may have less cheliceral bite force than hunting spiders [80]. In terms of web-building spiders, orb webs are more efficient at capturing small prey compared to tangle or sheet webs [81] as more prey are intercepted and fail to escape. And large orb-webs are well adapted to maximize prey capture [82]. For web-building spiders, diet composition and trophic interactions depend in part on the habitat where webs are built [83], web design [70], and body size [84]. In addition, the composition of web silk may differ not only between species [67], but by individuals within a species in relation to prey availability [85]. Further research on diets outside of agricultural systems with attention to other factors that influence prey capture will help clarify the trophic structure of more spider species. This is especially important for native spiders that may overlap with introduced or invasive predators.

The combination of DNA metabarcoding and high-throughput sequencing are relatively new approaches to study trophic niche overlap and diet competition. Based on the nature of the sequence data generated, how analytical decisions effect dietary patterns, such as modeling relative read abundance versus frequency of occurrence [86] as well as multivariate model convergence [62] and taxonomic resolution are still being ironed out. As such, few metabarcoding studies have addressed the effects of taxonomic resolution on patterns of dietary niche overlap [but see 7,11]. For many predators, such as spiders, taxonomic resolution of prey is often poor at the genus or species level and depends on sample size [74]. For example, in a web-building spider *Araneaus diadematus*, detection of approximately 90% of prey orders can be achieved with a sample size of less than 10 individuals, however, sample size estimates for 90% resolution at the species level likely requires greater than 100 individuals [74]. Furthermore, the number of samples needed to detect approximately 90% of prey diversity at any given level of classification is influenced by the foraging behaviors of individual spider species, such hunting style and diel activity patterns [74]. Despite these differences, for spiders, molecular tools have significantly advanced our understanding of foraging ecology [87]. Spiders, because they are extra-intestinal liquid feeders, cannot be dissected under a microscope to identify prey fragments [22]. Thus, non-molecular approaches to determine trophic niche overlap, which rely on morphology-based fragment analysis of partially digested prey are not possible. Moreover, morphology-based identification is often limited to family level classifications. Despite the limitations, molecular gut content analysis, particularly for spiders, is highly advantageous in terms of moving the field forward to understand community dynamics [87].

Despite many advantages, there are inherent limitations to molecular gut content for analysis of arthropod food webs. For example, frequency of occurrence is based on the presence or absence of prey taxa and does not take into account the number of prey consumed [86]. As such, dietary overlap among our focal species is based on presence or absence alone. In addition, due to the taxonomic assignments that provide the best possible dataset at the family level (i.e., inclusion of the greatest proportion of reads), we likely underestimate specific species associations. This would also alter the diet overlap metrics and estimation of dissimilarity. Therefore, the combined set of various diet metrics provide a conservative estimate of diet overlap and dissimilarity, which still points to unique diets in the Joro and native spiders. Furthermore, due to unknown quantities of prey detected in spiders or webs, observation of foraging patterns on the web are needed to confirm the proportion of various prey taxa in the diets of each species. In addition, for spider metabarcoding studies, focal predator reads often constitute a significant proportion of the total reads, which may impact estimates of prey richness [88]. To address this, we amplified our samples with a spider exclusion primer, designed to reduce the amount of spider DNA [52], however, a large proportion of our total reads amplified Araneae DNA for each focal spider (Table 1). And finally, because DNA metabarcoding and high-throughput sequencing is extremely sensitive, we are likely detecting secondary feeding events or tri-trophic interactions [89]. Due to the nature of sample collection, we are unable to discriminate between direct consumption and secondary feeding.

## Conclusions

Globalization has facilitated the movement of non-native species, and over time, new introductions are projected to increase [90]. Particularly for arthropods, trade routes are a primary source of introductions [91], which is presumably how Joro spiders arrived in the United States [32]. Once established, Joro spiders become locally abundant and may serve as a new source food for native vertebrate predators, such as birds [92,93]. Joro spiders may also contribute to biological control of pest and nuisance insects, such as stink bugs, mosquitoes, and flies. However, many speculate that Joro spiders may outcompete native orb-weaving spiders [34,48]. Yet, we currently lack much of the necessary life history information and prior population size estimates for native spiders that live in habitats where non-native Joro spiders are now found. Here, we take an initial step to understand the diets of Joro spiders and co-occurring, orb-weaving spiders. Overall, Joro and native spider diets are unique, and the presence of Joro spiders did not change the composition of native spider diets. Therefore, it is unlikely native orb-weaving spider will be displaced by Joro spiders based on diet competition alone. Nevertheless, more study is needed regarding native spiders before we can conclude the probability of displacement by Joro spiders or negative impacts on their populations.

## Supporting information

S1 File This file contains supplemental figures. (DOCX)

S1 Table Table displays the number of individuals with a given prey taxa detected in spider gut samples, where counts are organized by focal spider species. A positive detection occurred when at least 21 reads were present for a given prey taxa by individual spider.
(XLSX)

S2 Table Represents the rank abundance of taxa detected in all spider samples (A) and total reads recovered from samples for the most common 52 taxa detected in spider DNA samples (B). Shaded area taxa are to emphasize the number of dipterans detected. The differences in rank between the two methods may be that more individuals of the taxa were consumed, and frequency only accounts for presence or absence.
(XLSX)

S3 Table Indicator species analysis output. (XLSX)
