# Supplementary material for: DNA metabarcoding to estimate diet overlap between the introduced Joro spider (Trichonephila clavata) and three native orb-weaving spiders
Source: PLoS One. 2026 Jun 24;21(6):e0351929. doi: 10.1371/journal.pone.0351929 (PMC13293469; doi:10.1371/journal.pone.0351929)
Supplement: S1 File — (DOCX) [file pone.0351929.s001.docx]

**Supporting Information**

**Title:** DNA metabarcoding to estimate diet overlap between the introduced Joro spider (*Trichonephila clavata*) and three native orb-weaving spiders

**Authors:** Erin E. Grabarczyk^1^ & Jason M. Schmidt^2^

**Affiliations**

^1^ Department of Biology, Valdosta State University, Valdosta, GA, USA. ORCID: 0000-0002-7659-8366

^2^ Department of Entomology, University of Georgia, Tifton, GA, USA.

* Corresponding authors: EE Grabarczyk email: egrabarczyk@valdosta.edu; JM Schmidt, email: jmschmid2@uga.edu

**Keywords:** DNA metabarcoding; high-throughput sequencing; molecular gut content analysis; predator-prey interactions; resource competition; niche partitioning

**S1 Fig. Map of collection sites**. Joro and native orb-weaving spiders were collected from 52 sites in Georgia and North Carolina, USA between October 2 – 24, 2021. Joro spiders (*Trichonephila clavata*) were present at collection sites indicated with a pink circle and absent from sites indicated with a green circle. Map imaged was generated in ArcMap 10.5 (Esri, Redlands, California, USA) using the USDA National Agricultural Statistics Service Cropland Data Layer (2022) and GPS locations of study sites.

**S2 Fig. Dissimilarity and centroid comparison between focal spider species.** (A) NMDS of the dissimilarity in prey DNA detection for focal spider gut samples. Dissimilarity was calculated on a DNA detection matrix using the Jaccard index and fit multiple times to reduce localized patterns using metaMDS, then scores were exported and plotted with ggplot2 (Oksanen et al. 2025, Wickham 2016). Results indicated diet composition was dissimilar between spider species (PERMANOVA, F_3,75_ = 3.8, *P* = 0.001; (B) Centroid comparisons of pairwise dissimilarity of prey DNA detection between spiders with 95% confidence limits around the mean, which is indicated at the center of the CI. Estimated using the function “betadisper” in the vegan R package (Oksanen et al. 2025). Labels for spider taxa: Joro = *Trichonephilia clavata*, Am = *Araneus marmoreus*, Neo = *Neoscona crucifera*, Gc = *Gasteracantha cancriformis*.

**A B**

**C**

**S3** **Fig.** **Dissimilarity and centroid comparison of native spiders collected from sites with and without Joro spiders.** (A) NMDS to show the dissimilarity in prey DNA detection patterns of native spiders collected from where Joro spiders were also detected (yes, green triangles) or not detected (no, yellow circles). Dissimilarity was calculated on the DNA detection matrix using the Jaccard index and fit multiple times to reduce localized patterns using metaMDS, then scores were exported and plotted with ggplot2 (Oksanen et al. 2025, Wickham 2016). Prey DNA taxa detected does not significantly correlate with the presence or absence of Joro spiders at collection sites (PERMANOVA, F_1,52_ = 1.3, *P* = 0.1); (B) standard box and whisker plots on centroid comparisons on distance of prey DNA detection between spiders, and (C) comparison of mean centroid difference plotted with a 95% confidence interval (Estimated using the function “betadisper” in the vegan R package (Anderson et al. 2006, Oksanen et al. 2025).

References

Anderson, M.J., Ellingsen, K.E. & McArdle, B.H. (2006) Multivariate dispersion as a measure of beta diversity. Ecology Letters **9**, 683–693.

Oksanen J, Simpson G, Blanchet F, Kindt R, Legendre P, Minchin P, O'Hara R, Solymos P, Stevens M, Szoecs E, Wagner H, Barbour M, Bedward M, Bolker B, Borcard D, Borman T, Carvalho G, Chirico M, De Caceres M, Durand S, Evangelista H, FitzJohn R, Friendly M, Furneaux B, Hannigan G, Hill M, Lahti L, Martino C, McGlinn D, Ouellette M, Ribeiro Cunha E, Smith T, Stier A, Ter Braak C, Weedon J (2025). _vegan: Community Ecology Package_. doi:10.32614/CRAN.package.vegan <https://doi.org/10.32614/CRAN.package.vegan>, R package version 2.7-1, <https://CRAN.R-project.org/package=vegan>.

United States Department of Agriculture (USDA) National Agricultural Statistics Service (NASS), 2022, Cropland Data Layer: USDA NASS, USDA NASS Marketing and Information Services Office, Washington, D.C. Online Links: <https://croplandcros.scinet.usda.gov/>

Wickham, H. ggplot2: Elegant Graphics for Data Analysis. Springer-Verlag New York, 2016.
